# A Normative Theory of Forgetting: Lessons from the Fruit Fly

**DOI:** 10.1371/journal.pcbi.1003640

**Published:** 2014-06-05

**Authors:** Johanni Brea, Robert Urbanczik, Walter Senn

**Affiliations:** 1 Department of Physiology, University of Bern, Bern, Switzerland; 2 Department of Physiology and Center for Cognition, Learning and Memory, University of Bern, Bern, Switzerland; Brain and Spine Institute (ICM), France

## Abstract

Recent experiments revealed that the fruit fly *Drosophila melanogaster* has a dedicated mechanism for forgetting: blocking the G-protein Rac leads to slower and activating Rac to faster forgetting. This active form of forgetting lacks a satisfactory functional explanation. We investigated optimal decision making for an agent adapting to a stochastic environment where a stimulus may switch between being indicative of reward or punishment. Like *Drosophila*, an optimal agent shows forgetting with a rate that is linked to the time scale of changes in the environment. Moreover, to reduce the odds of missing future reward, an optimal agent may trade the risk of immediate pain for information gain and thus forget faster after aversive conditioning. A simple neuronal network reproduces these features. Our theory shows that forgetting in *Drosophila* appears as an optimal adaptive behavior in a changing environment. This is in line with the view that forgetting is adaptive rather than a consequence of limitations of the memory system.

## Author Summary

The dominant perception of forgetting in science and society is that it is a nuisance in achieving better memory performance. However, recent experiments in the fruit fly show that the forgetting rate is biochemically adapted to the environment, raising doubts that slower forgetting *per se* is a desirable feature. Here we show that, in fact, optimal behavior in a stochastically changing environment requires a forgetting rate that is adapted to the time constant of the changes. The fruit fly behavior is compatible with the classical optimality criterion of choosing actions that maximize future rewards. A consequence of future reward maximization is that negative experiences that lead to timid behavior should be quickly forgotten in order to not miss rewarding opportunities. In economics this is called “minimization of opportunity costs”, and the fruit fly seems to care about it: punishment is forgotten faster than reward. Forgetting as a trait of optimality can further explain the different memory performances for multiple training sessions with varying inter-session intervals, as observed in a wide range of species from flies to humans. These aspects suggest to view forgetting as a dimension of adaptive behavior that is tuned to the environment to maximize subjective benefits.

## Introduction

*Drosophila melanogaster* forgets [1], [2]. In itself this is unremarkable because forgetting as a behavioral phenomenon appears in any adaptive system of limited capacity; storing new associations will lead to interference with existing memories. Forgetting, in this sense, is just the flip side of learning. When capacity is not an issue, forgetting may nevertheless be caused by a useful mechanism: one that keeps a low memory load and thus prevents a slowdown of retrieval [3], [4]. Consequently, capacity or retrieval limitations lie at the heart of standard theories of non-pathological forgetting [5], [6], which focus on interference and decay explanations. Alternatively, forgetting has been proposed to be an adaptive strategy that has evolved in response to the demands of a changing environment [7]. It is the latter explanation that seems to apply to *Drosophila* where the experimental evidence suggests that the cause underlying forgetting is an active process which is modulated by the learning task and not by internal constraints of the memory system; in particular in olfactory conditioning tasks, reversal learning leads to faster forgetting [8] whereas spaced training leads to slower forgetting compared to single or massed training [9]. Further, forgetting in *Drosophila* seems rather idiosyncratic in that aversive conditioning is forgotten approximately twice as quickly as appetitive conditioning [10], [11].

In psychology, the term forgetting commonly refers in “to the absence of expression of previously properly acquired memory in situations that normally cause such expression.” ([6]; see also [12]). Similarly, in conditioning experiments, one speaks of forgetting, when the conditioned stimulus fails to evoke the conditioned response at some point after successful conditioning [8], [13].

**Figure 1:**
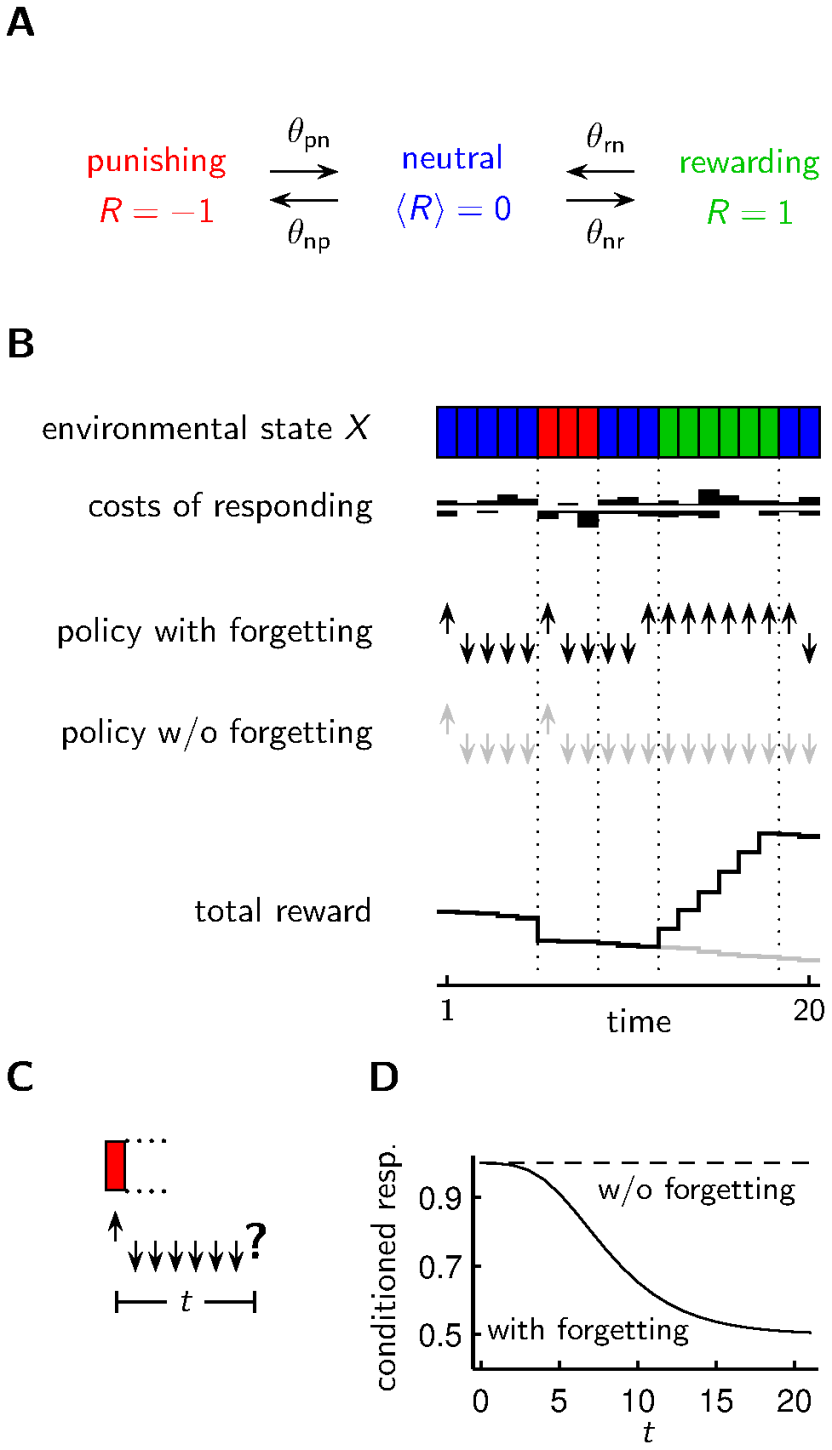
Agent acting in a changing environment. **A** The environmental state changes stochastically with rates 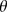 between being rewarding, neutral or punishing. Unless mentioned otherwise, we choose 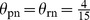 and 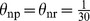. **B** Based on a policy (with forgetting, without forgetting) which may depend on past observations of the environmental state and current costs of responding, an agent shows the appetitive reaction (upward arrow) or the aversive reaction (downward arrow). The stochastic costs (i.i.d. with an exponential distribution with scale parameter 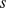) for the appetitive/aversive reaction are shown above/below the white line. An agent with a policy that involves forgetting accumulates more reward than an agent without forgetting or immediate forgetting. **C** In an emulation of a classical conditioning experiment, the agent experiences a defined environmental state, and after a waiting period of length 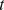 the agent has to react according to the internal policy. **D** Different policies lead to different outcomes in classical conditioning experiments. Shown is the fraction of agents choosing the conditioned response (conditioned resp.) at time 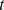 after conditioning for agents subject to individual costs of responding.

In the basic protocol for behavioral studies of memory in *Drosophila* [1] a group of flies is placed into a tube for conditioning. There the flies are exposed to a specific odor and the exposure is paired with a reinforcer (sugar or electrical shock). Having experienced the pairing once or multiple times, the flies are removed from the conditioning tube. After a predefined delay time, the group is placed into the middle of a second, elongated tube for assessment. One side of the elongated tube is baited with the conditioned odor and, after a while, the fraction of flies is determined which exhibit the conditioned response by comparing the number of flies which are closer to the baited side of the tube with the number of flies closer to the un-baited side. The setup allows to measure memory performance (c.f. Fig. 1 D), i.e. expression of the conditioned response, as function of the delay time and of the conditioning protocol (e.g. magnitude of reinforcement, number of pairings). To check for bias in the setup, one typically in addition uses a second odor as a control which was not paired with a reinforcer.

That *Drosophila* has a dedicated mechanism to control forgetting was convincingly demonstrated by Shuai et al. [8] and Berry et al. [2]. Inhibition of the small G-protein Rac leads to slower decay of memory, extending it from a few hours to more than one day [8]. Conversely, elevated Rac activity leads to faster forgetting [8]. Similar results were achieved by modulation of a small subset of Dopamine neurons [2]. Stimulating these neurons leads to faster forgetting after aversive and appetitive conditioning, while silencing these neurons leads to slower forgetting [2].

Given the importance of decision making, it appears unlikely that forgetting in *Drosophila* is a behavioral trait which is maladaptive in an ecological sense. Hence we investigated what generic model of the environment would justify the observed forgetting and in particular the asymmetry between aversive and appetitive conditioning. For this we mathematically determined optimal decision making strategies in environments with different associations between stimulus and reinforcement.

## Results

### Basic model of decision making in a changing environment

For our model we assumed a simplified scenario where the conditioning pertains directly to the appetitive reaction. In particular, depending on the state of the environment, approaching the odor can lead to reward (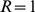) or punishment (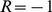) but it can also result in no reinforcement (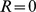) (Fig. 1). Fleeing the odor, i.e the aversive reaction, never leads to reinforcement (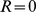). An agent (fruit fly), whose goal is to maximize reinforcement, chooses between the appetitive and aversive reaction depending on past experience. To model the non-deterministic behavior observed in the experiments we assume that the two available behavioral options involve different costs of responding. These costs of responding, however, fluctuate from trial to trial causing no bias on average. For instance, a fly which happens to find itself to the right of the group initially could well have a smaller cost of responding for staying on this side of the assessment tube on this trial. More generally, the stochastic costs of responding can be seen as incorporating all other factors that also influence the behavior but do not depend on the past experiences that involve the conditioned stimulus. The total reward received by the agent is the external reinforcement (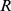) minus the cost of responding. Our agent takes this into account in decision making, and so the costs of responding result in trial to trial fluctuation in the behavior. Whether the appetitive reaction results in 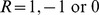 depends on the state of the environment. This state changes slowly over time (according to a Markov chain, see Methods and Fig. 1A). So when the appetitive reaction results in 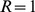 on one trial, the same outcome is likely on an immediately subsequent trial, but as time goes by the odds increase that the appetitive reaction results in 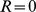 or even punishment.

### The agent maintains a belief about the environmental state

**Figure 2:**
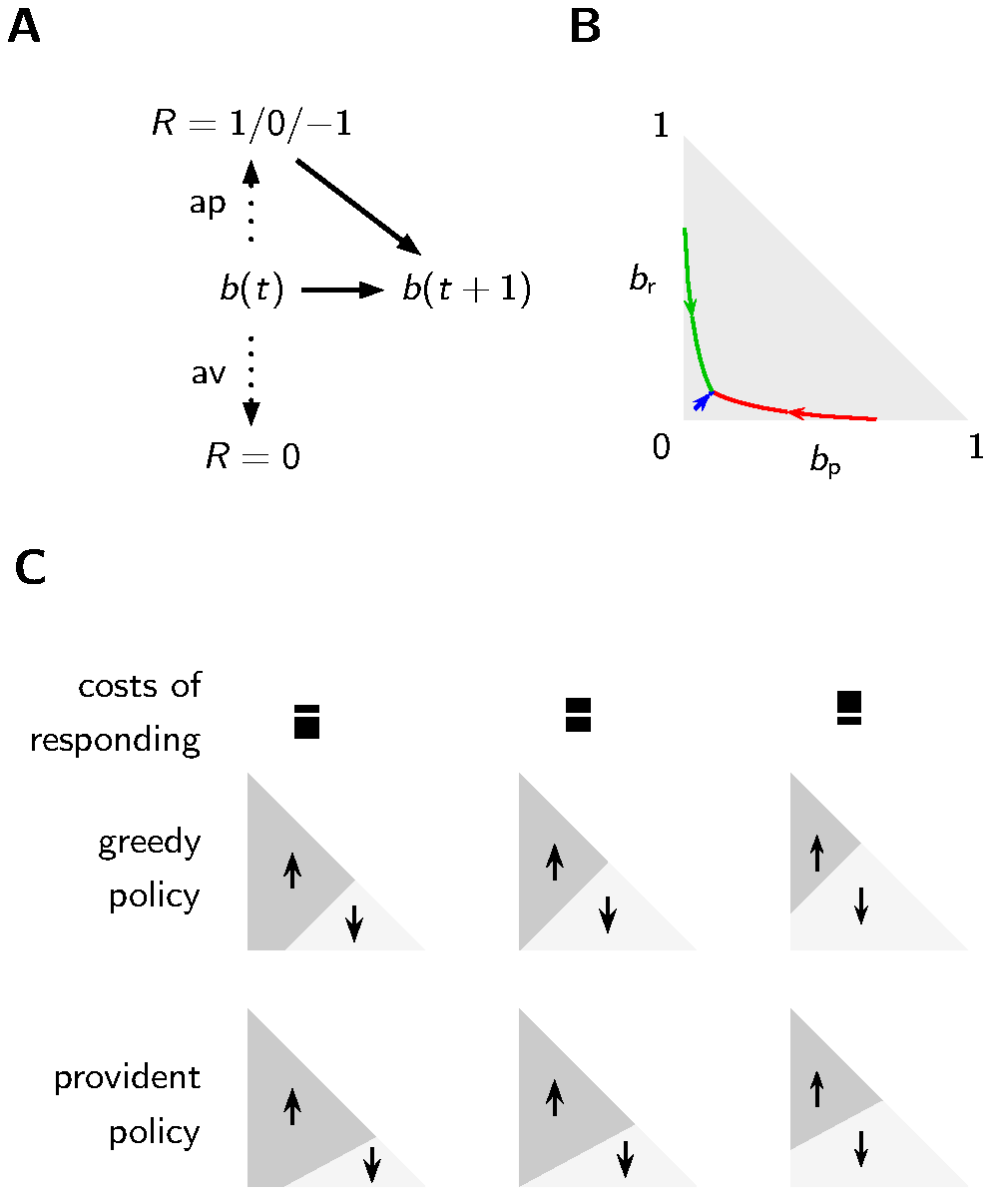
Belief and policy of an agent acting in a changing environment. **A** The belief about the environmental state 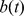 may influence the choice of the appetitive or aversive reaction. Only after the appetitive reaction, the agent gains new information about the true state of the environment. The belief 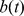 and the agents knowledge about the transition probabilities of the environmental state combined with potentially new information determines the new belief 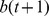. **B** The starting point of the arrows is a belief found by choosing the appetitive reaction once and receiving reward (green), punishment (red) or no reinforcement (blue). If the agent always chooses the aversive reaction thereafter, the belief drifts to the stationary state along the trajectories shown by the arrows. Possible belief states 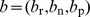 with 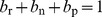 can be represented as a point in the “belief space” (gray shaded triangle). **C** The regions in the belief space favoring the appetitive reaction (dark shading, upward arrow) over the aversive reaction (bright shading, downward arrow) depend on the policy and the costs of responding. The provident policy (lowest row) is biased towards the appetitive reaction. A larger cost for the aversive reaction than for the appetitive reaction (left column) decreases the region of the aversive reaction.

If the agent knew the environmental state, the best policy would be simple: choose the appetitive (aversive) reaction if the environmental state is rewarding (punishing). Typically however, the agent does not know the actual environmental state but, at best, maintains a belief about it (see Fig. 2A and Methods). In our model, the belief consists of the probabilities 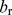, 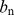 and 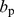 to receive rewarding, neutral or punishing reinforcement, respectively, after selecting the appetitive reaction. Geometrically, the belief can be represented as a position in a 2-dimensional belief space that stepwise changes after the appetitive reaction and thus gaining new information about the current environmental state and otherwise drifts towards an equilibrium (forgetting), see Fig. 2B (note that, since the three probabilities sum to one, the probability of the neutral state can be computed given the probabilities of the rewarding and punishing state, i.e. 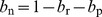).

If e.g. a fly gets punished, the probability 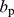 to be punished again on the next trial is high (initial point of red trajectory in Fig. 2B). If subsequently the fly chooses the aversive reaction, the belief will drift towards a stationary value (end point of red trajectory in Fig. 2B). We assume that the agent has implicit knowledge, e.g. gathered by experience or through genetic encoding, about the transition rates of the environmental state.

### Acting according to a greedy policy leads to forgetting

Based on belief values and costs of responding one may define different policies. A greedy policy selects the appetitive reaction if the agent believes that reward is more probable than punishment and costs of responding are equal for both actions, i.e. 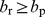 (Fig. 2C top, middle). If costs for one reaction are larger than for the other, the region in the belief space favoring this higher-cost reaction becomes smaller (Fig. 2C top, left and right). Immediately after conditioning, an agent has a strong belief that the environment is still in the same state as during conditioning. Thus, if the greedy policy determines action selection, an agent most likely chooses the conditioned response. As the belief drifts towards the stationary point, the stochastic costs of responding gain more influence on the decision making and thus an agent is more likely to have already forgotten the conditioning, i.e. the agent is more likely to choose the opposite of the conditioned response. We call this policy “greedy”, because it maximizes reward if only one choice is made but it is not necessarily optimal with respect to gaining future rewards. Technically, the greedy policy is equivalent to the optimal future discounted policy with discount factor 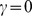, i.e. the policy that neglects future rewards.

### Dependence of the forgetting curve on parameter choices

**Figure 3:**
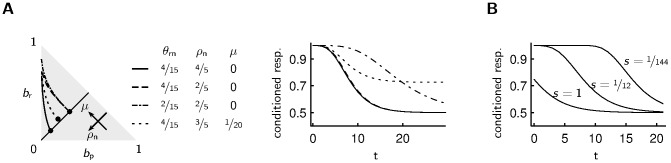
Dependence of the forgetting curves on the model parameters. **A** Left: The stationary belief state in the absence of observations (indicated by dots) moves along the direction of the arrows for increasing probability of the neutral state 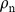 or increasing average reward 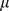. How fast the belief drifts towards the stationary state after receiving reward depends on the parameter 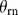 that controls the “timescale of changes”. Right: Changing the probability of the neutral state 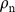 only marginally affects the forgetting curve (solid and dashed line). A smaller rate of changes 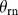 leads to slower forgetting (dash-dotted curve). A positive average reward 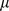 leads to a higher fraction of agents choosing the appetitive reaction, which is here the conditioned response (dotted curve). **B** For a large variance of costs of responding (curve with scale parameter of the exponential distribution 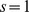) there are some agents that do not exhibit the conditioned response immediately after conditioning, since the costs of the conditioned response are too large. If the variance of the costs of responding is small (curve with 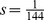), most agents choose the conditioned response until their belief gets close to the stationary belief state.

In order to conveniently analyze the forgetting behavior under the greedy policy for different choices of the environmental parameters 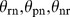 and 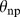 (Fig. 1A), we use a re-parametrization with the “probability of the neutral state” 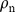 and the “average reward” 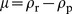, where 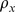 denotes the stationary state probability of state 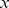 (see Methods for the relationship between 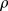 and 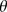). Changing the probability of the neutral state 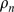 has almost no effect on the forgetting curve (Fig. 3A, solid vs. dashed line). Increasing the average reward has the consequence that in the stationary state more agents select the appetitive reaction than the aversive reaction (Fig. 3A, solid vs. dotted line). The speed of forgetting a conditioned state (p, n or r) is determined by the rate of transitioning away from this state. Fig. 3A (solid vs. dash-dotted line) shows the effect of changing the rate 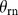, whose inverse is equal to the average number of timesteps the environment spends in the rewarding state: forgetting is faster for a larger rate 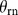. The variance of the costs of responding determines the impact of the costs of responding on decision making. For large variance the forgetting curve is closer to 0.5 than for small variance, since for large variance it is more likely that the costs of responding have a strong impact on decision making (Fig. 3B).

### Acting according to a provident policy leads to faster forgetting after aversive conditioning than after appetitive conditioning

**Figure 4:**
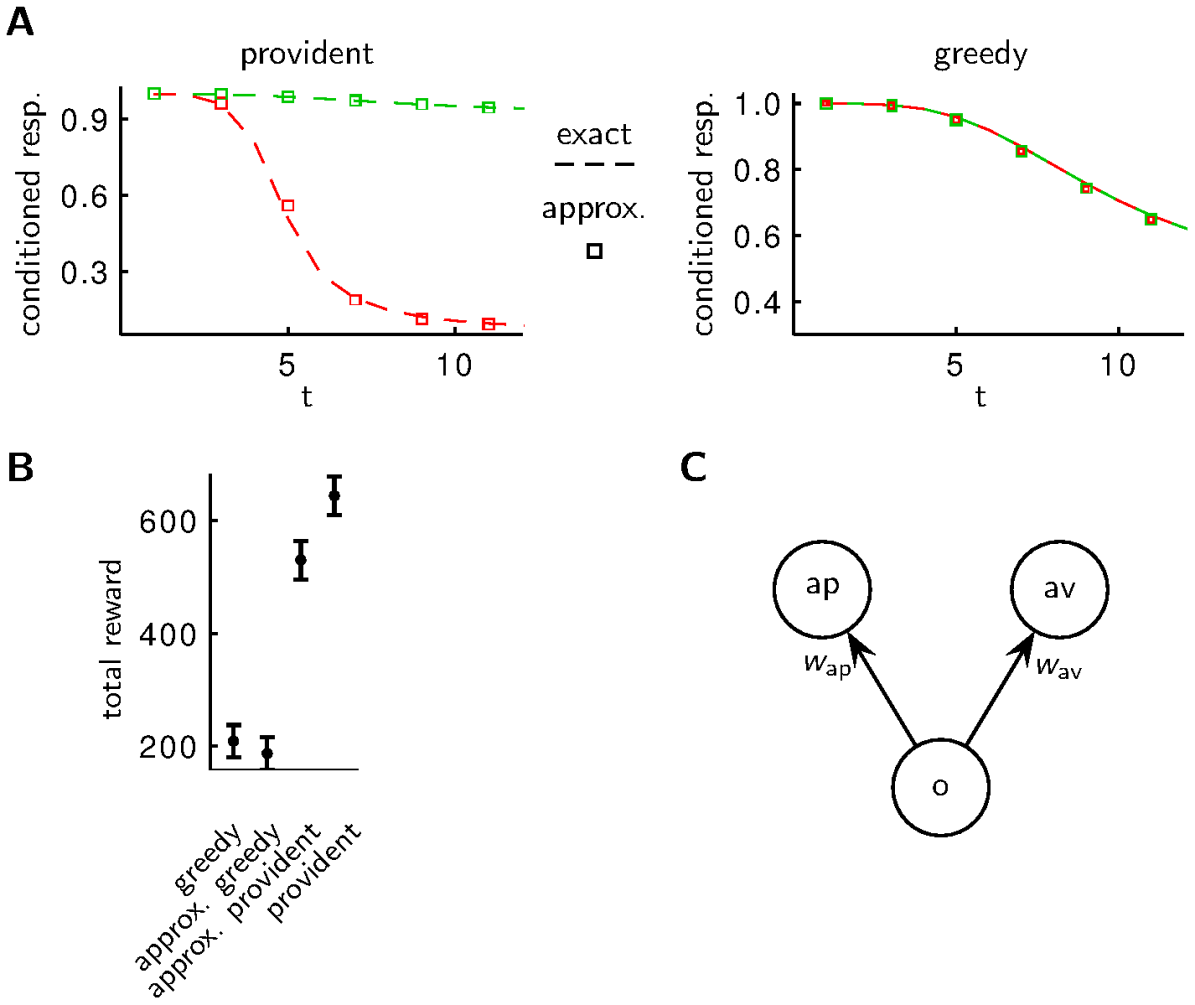
Asymmetry of behavior after aversive and appetitive conditioning. **A** An agent with a provident policy shows faster forgetting after aversive conditioning (red curve) than after appetitive conditioning (green curve). The boxes mark the behavior of the approximative model in C. **B** The total reward collected in free runs of 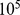 time bins (compare to Fig. 1B) is larger for the provident policy than for the greedy policy. Plotted are mean and s.e.m. for 40 trials. **C** Similar performances are obtained with a simple, approximative implementation of the optimal strategy with synaptic strengths 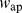 and 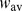 connecting an odor detecting neuron (o) to action neurons “approach” (ap) and “avoid” (av). In the absence of any stimulus (odor) the synaptic strengths decay with different time constants for the approximative provident policy and with the same time constants for the approximative greedy policy. When an odor is present, the synaptic strengths change in a Hebbian way in the case of reward and in an anti-Hebbian way in the case of punishment, i.e. 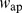/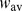 increase/decrease for reward and decrease/increase for punishment.

While the difference in forgetting speed after appetitive and aversive forgetting could be a consequence of different transition rates 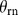 and 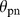, such a difference also arises if these rates are equal but the agent uses a provident policy, i.e. a policy that also takes into account future rewards. In the long run the provident policy is superior to the greedy policy (Fig. 4B). We therefore determined numerically a policy which approximately maximizes the reward rate, i.e. the total reward accumulated over a long period divided by the length of this period (see Methods). The resulting policy is such that there are beliefs for which the appetitive reaction is chosen, even when the probability of punishment is larger than the probability of reward, i.e. 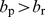, and the costs of responding are equal for both actions (Fig. 2C bottom, middle). The reason for this becomes clearer when we look at what economists call the opportunity cost, i.e. the additional gain that has not been harvested because of missing to choose the (often by hindsight) better option [14]. For the appetitive reaction, the agent's opportunity cost is given by the potentially lower cost for the aversive reaction. But for the aversive reaction, the agent's opportunity cost is not only the potentially lower cost for the appetitive reaction but also the lack of further information about the actual environmental state. This information is required for best exploitation in future trials. Assume, for instance, that at some point in time the agent believes that punishment is slightly more probable than reward and therefore sticks to the aversive reaction. Now, if the actual environmental state would be rewarding, the agent would not only miss the current reward but also misses subsequent rewards that could potentially be harvested while the state is still rewarding. When taking this opportunity cost into account, the agent will choose the appetitive reaction despite the belief state slightly favoring the aversive reaction. For an external observer this optimal choice behavior appears as a faster forgetting of the aversive memory. In short, the asymmetry in forgetting after aversive and appetitive conditioning (Fig. 4) arises because choosing the appetitive reaction is always informative about the current environmental state whereas choosing the aversive reaction is not.

### A simple mechanistic implementation results in close to optimal behavior

The probabilistic calculations needed to derive the optimal provident behavior can be quite involved. We do not suggest that there is a neuronal circuitry in *Drosophila* which actually does these calculations. Yet it is interesting to note that a much simpler mechanistic decision making model already results in close to optimal behavior (Fig. 4B). This simple model allows an interpretation of the variables as synaptic strengths from odor sensitive neurons to decision neurons (Fig. 4C). In the absence of odor and behavioral feedback the synaptic strengths decay with different time scales towards a stationary level: decay is faster for synapses targeting the “avoid” neurons than for the “approach” neurons. One could speculate that the speed of this decay is governed by e.g. the concentration of Rac [8] or dopamine [2].

### *Drosophila* adapts to changing environmental time scales

So far we have assumed that the transition rates between the environmental states are fixed. This is not an assumption *Drosophila* seems to make and in fact, would be an unrealistic model of the environment. The experiments by Tully et al. [9] show that forgetting depends not only on the number of conditioning trials but also on their frequency. In particular, forgetting is slower when the same number of learning trials is spaced out over a longer period of time. Spaced training is more informative about the environment being in a slowly changing mode than the temporally compressed massed training. Furthermore, reversal training during which in fast succession an odor is aversively, neutral and again aversively conditioned [8] results in faster forgetting and is informative about a fast changing environment. So the observed behavior provides rather direct evidence that adaptation in *Drosophila* does indeed take non-stationarity into account.

### Extended model with slow and fast transitions matches the observed behavior for different conditioning protocols

**Figure 5:**
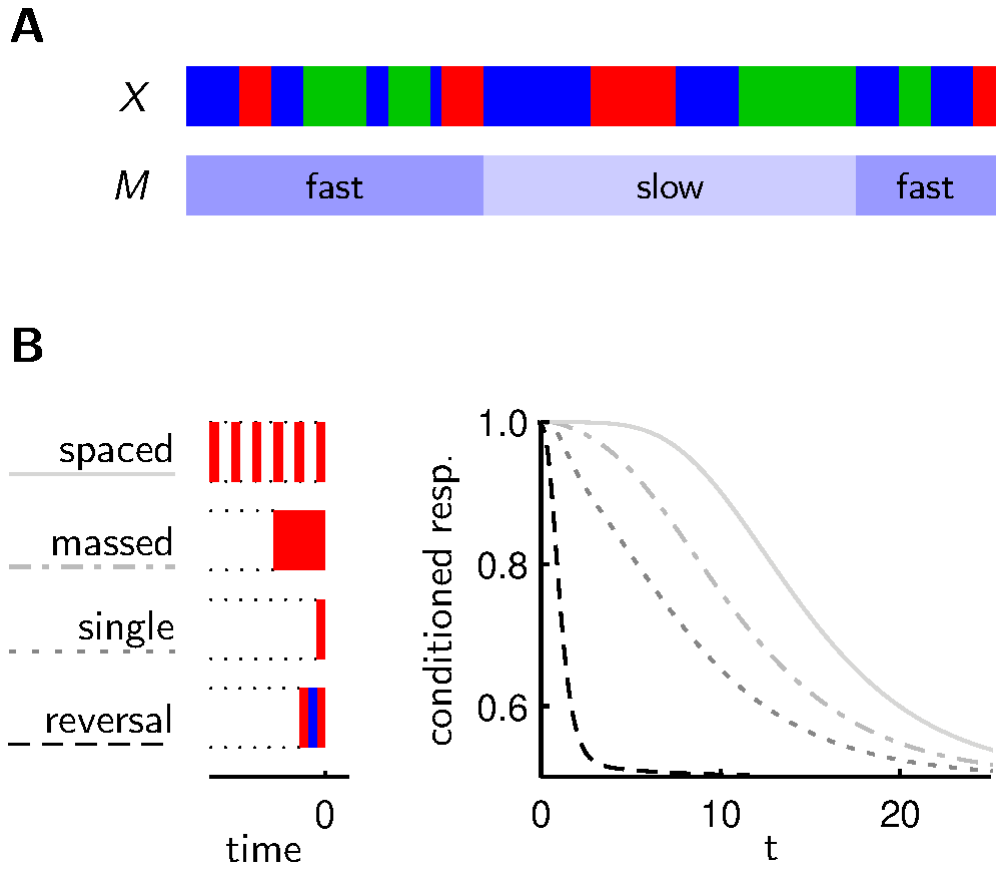
Behavior of agents that estimate the time scale of non-stationarity. **A** In an extended model the rate of change depends on a slowly changing meta variable 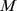, which can be in a slow or fast state. **B** As observed in experiments with *Drosophila*, our model agents show slowest forgetting after spaced training and fastest forgetting of the last association after reversal training. In our model, this result appears as a consequence of spaced training being most informative about slow transitions, whereas reversal training is most informative about fast transitions.

To include adaptation as a response to changing transition rates, we extended our model by a slowly varying meta variable 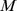 which can either be in state “fast change” or “slow change” (Fig. 5A). The dynamics of the meta variable 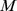 is governed by a Markov process with small transition rates. In state “fast change”, the environmental reward state 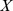 changes more rapidly than in state “slow change”. In this setting, an optimal agent maintains a belief about both the environmental reward state 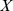 and the “hidden” state 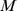 that sets the time scale of the changes in 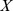. Spaced training increases the belief that the environment is in a slowly changing mode, whereas reversal learning leads to a strong belief about the environment being in the fast changing mode. The resulting greedy-optimal behavior is in qualitative agreement with the known behavior after spaced, massed and reversal learning (Fig. 5B) as observed for flies [8], [9], honey bees [15], pigeons [13], and humans [16].

## Discussion

We demonstrated that forgetting appears when an agent, subject to costs of responding, acts optimally in an environment with non-stationary stimulus-reinforcement associations. Based on reward maximization in a non-stationary environment, which is a reasonable objective not only for the fruit fly but for other species as well, our normative theory of forgetting includes an asymmetry in forgetting speed after aversive and appetitive conditioning and an adaptation of forgetting speed after spaced, massed and reversal learning. The asymmetry is the result of an economically optimal provident policy, which forages not only for immediate reward but also for information required for future exploitation. The adaptation of forgetting rate after spaced, massed and reversal learning is a consequence of the agents estimation of the current rate of environmental changes.

That costs of responding influence the action selection is an assumption which is in agreement with test-retest experiments [9], [11], [17]. In these classical conditioning experiment the flies are grouped according to whether they choose the conditioned response or not. Both groups are immediately retested to examine whether the flies stick to their decision. The outcome is: they do not. An equal fraction of flies chooses the conditioned response in both retest groups and this fraction is the same as in the first test containing all flies. This suggests that all flies maintain traces of the conditioning but that also other factors influence the choice in a stochastic way. Similarly, in our model the belief is a sufficient statistic of the past experiences that involve the conditioned stimulus and the stochastic costs of responding account for other factors that influence the choice.

A key assumption in our normative explanation of the differential forgetting in *Drosophila* is that the relationship between conditioned stimulus and reinforcement is non-stationary. Now, if this relationship were completely stationary, it would not need to be learned by the phenotype because it would already have been learned by the genotype, i.e. in this case the stimulus would be an unconditioned stimulus. Hence, from an evolutionary perspective, our assumption is close to being a truism. Nevertheless, many biological models of reinforcement learning have, for the sake of simplicity, assumed a stationary stimulus-reinforcement relationship [18], [19].

Experiments and models with non-stationary stimulus-reinforcement associations have suggested, similar to our findings, that in a more volatile environment the learning should be faster [20]–[24]. However, faster learning does not unconditionally imply faster forgetting. The asymmetry in forgetting speed after appetitive and aversive conditioning additionally requires an evaluation of the behavioral relevance of a specific memory content. Since the aversive reaction is not informative about the current state of association, aversive conditioning should be forgotten faster than appetitive conditioning.

Finding the optimal policy in an environment with a non-stationary stimulus-reinforcement relationship, as considered here, is computationally involving. As we have shown, however, approximately optimal decision making is still possible with a simplified neuronal model using experience induced synaptic updates. This model incorporates forgetting in the decay time constant of the synaptic strengths. As the parameters describing the changing environment are assumed to be constant across generations, the neuronal architecture and the forgetting rates can be considered to be genetically encoded.

Since the work of Ebbinghaus [25] on the forgetting rate of non-sense syllables and the observation of Jenkins and Dallenbach [26] that sleep between learning and recalling reduces forgetting, cognitive psychologists debate about the role of natural decay and interference in explaining forgetting [5]. While interference based explanations are favored by many [5], [12], Hardt et al. [6] recently advocated active processes behind decay-driven forgetting. They suggested a memory system that engages in promiscuous encoding and uses a flexible mechanism to remove irrelevant information later, mostly during sleep phases. In their view, different forgetting rates are a sign of such a flexible removal mechanism. But why do biological organisms need to actively remove irrelevant memories at all? Popular answers so far implicitly assumed that forgetting is ultimately the result of some limitation of the memory system, for instance, limited storage capacity, a limit on the acceptable read-out time for the memory or a decay of the biological substrate similar to unused muscles atrophy [6], [27]. In our model, however, forgetting does not result from a memory limitation, but emerges as an adaptive feature of the memory system to optimally cope with a changing environment while accounting for the relevance of different memory contents.

## Methods

### Basic model of the environment

In time bin 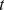 an odor can be associated with one of three environmental states: 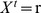 (reward), 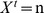 (neutral), 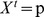 (punishment). The time-discrete dynamics of the environmental state is given by a Markov Chain with state space 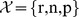 and transition probabilities 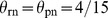 and 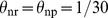, where 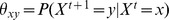 for 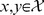. For simplicity we did not include direct transitions between the rewarding and punishing state, i.e. 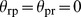. Including them would also allow for a behavior where the preference switches from the conditioned response to the opposite of the conditioned response before reaching the stationary state. The stationary distribution of this Markov chain, satisfying the self-consistency equation 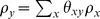, is given by 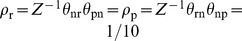 and 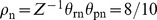, where 
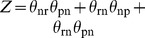
.

### External reinforcement signal

In each time bin 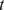 the agent has two behavioral options: approach the odor (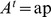) or avoid the odor (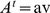). If the agent avoids, a neutral reinforcement signal 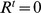 is always returned. If the agent approaches, the external reinforcement signal depends on the environmental state: there will always be a positive signal 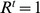 if 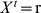, always a negative signal 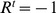 if 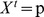 and if the odor is associated with the neutral state (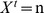), the agent will stochastically get a neutral signal 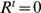 with probability 0.99, while with probability 0.005 the agent will get a positive or a negative reinforcement signal. Positive and negative reinforcement signals during the neutral state are included to model situations, where reward or punishment depends on odor unrelated factors. For further use we summarize the information in this paragraph in the probabilities 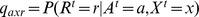, with non-zero entries 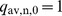, 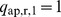, 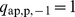 and 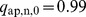, 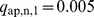, 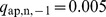.

### Belief

The agent maintains a belief 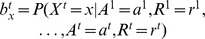 over the current environmental state 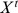 given past reinforcement 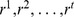 and actions 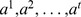. The belief state is updated by Bayesian filtering
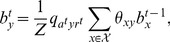
(1)with normalization 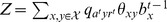. We use the abbreviation 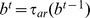 to denote the update of the belief 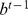 given action 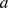 and reinforcement signal 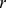.

### Costs of responding

We modeled costs of responding with exponentially distributed and uncorrelated random variables 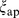 and 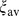 with parameter 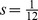, i.e. the probability density function of 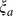 is given by 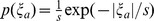 if 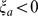 and 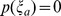 otherwise. This distribution has mean 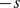 and standard deviation 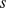. We assumed, that the agent receives an effective reward, which is a sum of the external reinforcement signal and the momentary cost of responding for the action chosen. During decision making, the agent knows the costs of responding for both actions but only has an expectation of the external reinforcement signal.

### Greedy policy: Maximization of immediate reward

If the goal is to maximize immediate reward, the agent's policy depends on the expected return in the next step 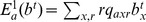, which for action ap can be simplified to 
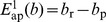
 and for action av is always zero, i.e. 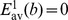. Including costs of responding, the policy that maximizes immediate reward selects the action for which 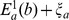 is maximal.

### Provident policy: Maximization of reward rate

A canonical choice of the objective to be maximized by a provident policy is the reward rate, i.e.
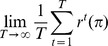
with expected reward 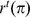 in time bin 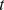 when acting according to policy 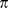. We approximately determined the policy which maximizes the reward rate by two methods: dynamic programming and linear programming on a quantized space.

Dynamic Programming allows to find a policy that maximizes the future discounted values
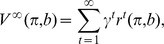
with discount factor 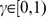 and expected reward 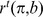 in time bin 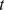 after starting in belief state 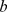 and acting according to policy 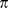. For finite state spaces and 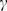 sufficiently close to 1 a policy that maximizes future discounted reward also maximizes the reward rate [28]. Without costs of responding one could directly apply the Incremental Pruning algorithm [29] to find a policy that maximizes the future discounted values. Here we derive dynamical programming in the presence of costs of responding.

Dynamic programming proceeds by iteratively constructing optimal finite-horizon values 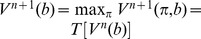 for some operator 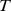. Assume that we have the horizon-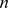 policy 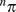 that maximizes the future discounted values of an episode of length 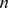. The horizon-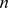 policy consists of instructions for each step in the episode 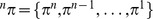, where 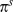 tells which action to take at the 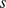-th step before the end of the episode, given belief 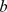 and costs of responding 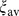 and 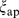. To construct the horizon-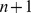 policy we need to extend the horizon-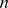 policy by the instruction for the first step, i.e. the 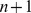-th step before the end of the episode. Without considering the costs of responding in the first step, the expected future discounted values for choosing action 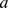 are given by
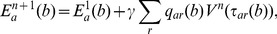
(2)where 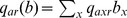 and the value function 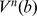 is given by (we will use the indicator function 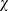, given by 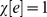 if 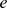 is true and 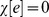 otherwise):
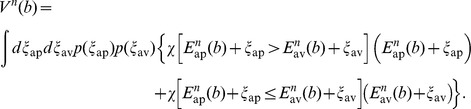



With the change of variables 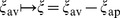, the resulting probability density function 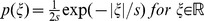 (Laplace probability density), and the abbreviations 
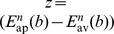
, 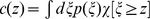 and 
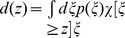
, we get
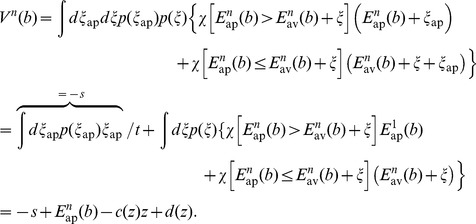
(3)


In the same manner we find the value function 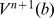, which depends through 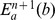 on 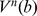 (see Eq. 2)
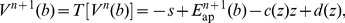
(4)where now 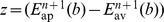.

Due to the discount factor 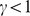 this recursion will eventually converge. In practice we will stop after 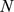 iterations and define the policy 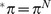, which approximates the future discounted policy. Note that in contrast to the finite horizon policies 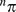 the policy 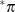 is stationary: in a sequential setting there is no end of an episode on which the policy may depend.

The number of terms in a naive implementation of 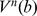 grows exponentially with 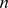. Without costs of responding the exponential growth can sometimes be prohibited by Incremental Pruning [29]. With costs of responding we are not aware of a way to prevent exponential growth. In Fig. 4 we approximated the stationary policy 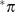 by taking the policy after 5 iteration with discount factor 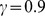, i.e. 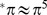. Since it is not clear whether for this choice of discount factor and number of iterations the resulting policy is a good approximation of the reward rate maximizing policy, we compared the result of dynamic programming with the policy obtained by linear programming on a quantized belief space.

For finite state and action space Markov Decision Processes linear programming can be used to find a policy that maximizes the reward rate [30], [31]. In our case, the policies act on the continuous space of belief states 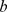 and cost of responding differences 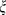. Analogous to the finite state space problem, the optimization problem could be formulated as: find functions 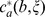 that implicitly define the policy [30] and satisfy
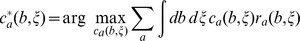


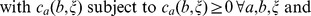


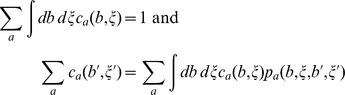
where 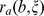 denotes the expected reward for action 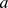, belief state 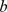 and costs of responding differences 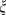 and 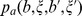 denotes the probability density to transition from 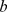 and 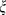 to 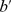 and 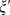 given action 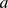. A straightforward approach is to quantize the belief space and space of cost of responding differences, replace the integrals by sums and find through linear programming an approximation to the reward rate maximizing policy. We quantized the two dimensional belief simplex 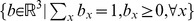 on a square lattice with different lattice spacings. Values that did not fall on lattice points where stochastically assigned to neighboring lattice points. The space of real valued cost of responding differences was quantized by segmenting the real line into adjacent intervals with equal mass of the probability density function. For each interval the average costs of responding for each action where computed. Using increasingly finer quantization we estimated the total reward to be between 600 and 655 for trials of 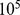 time bins, which is in agreement with the estimate obtained with dynamic programming (Fig. 4B provident).

### A simple, approximative implementation

In Fig. 4 we demonstrate that also an agent with two uncoupled low-pass filters can show near to optimal behavior. The agent's decision to approach (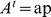) or avoid (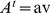) the odor depends on whether 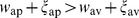, where 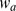 (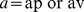) are variables interpretable as synaptic strengths and where 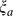 represents stochastic input due to costs of responding. The values of 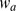 decay with different time-constants, in the case of no feedback, because the agent either stays away or no odor is present. If the agent approaches the odor and experiences reward, 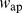 is set to a maximal value, while 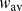 is set to zero; for odor plus punishment 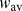 is set to a maximal value, while 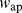 is set to zero. Formally, with the subscript 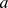 standing for either ap or av, we get
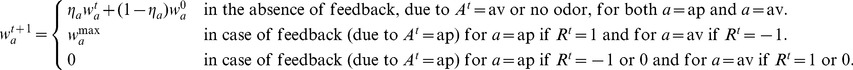
(5)The parameter 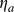 controls the speed of forgetting, 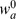 sets a baseline value and 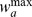 sets a maximum value. In Fig. 4 the parameter values where fit to the curves in sub-figure A (approx provident: 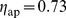, 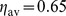, 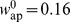, 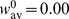, 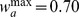) and to the curves in sub-figure B (approx greedy: 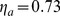, 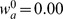, 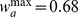).

### Extended model of the environment

To study the behavior of an agent that additionally has to estimate the rate of change we extended the basic model of the environment with a meta variable that controls the rate of change of the environmental state. In time bin 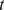 the meta variable can be in one of two states: 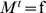 (fast) or 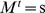 (slow). The dynamics of the meta variable is described by a Markov Chain with transition probabilities 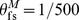 and 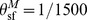. If the meta variable is in the slow (fast) state the transition parameters of the environmental state are 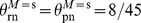, 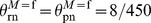 and 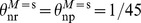, 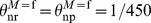. In the extended model the state space is given by the product space 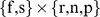 and the transition parameters are given by 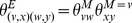. The agent maintains a belief about both the environmental state and the state of transition speed.

### Spaced, massed and reversal learning

In spaced training, the agent was aversively conditioned six times with intermittent waiting periods of 9 time bins. In massed training, the agent was aversively conditioned in 6 subsequent time bins. In reversal learning, the agent was exposed to the punishing, neutral and punishing environmental state in subsequent time bins. Forgetting curves are shown for the computationally less involving greedy policy. In order to compare massed with spaced training we choose a finer time discretization in the extended model, i.e. 10 time bins in the extended model correspond to 1 time bin in the basic model. In figure 5B the result is plotted in units of the basic model.
